# Anesthetic Management of a Patient With Parkinson’s Disease and a Deep Brain Stimulator Device for Hemiarthroplasty Surgery: A Case Report

**DOI:** 10.7759/cureus.41400

**Published:** 2023-07-05

**Authors:** Zainub Jooma

**Affiliations:** 1 Department of Anesthesia, Faculty of Health Sciences, School of Clinical Medicine, University of the Witwatersrand, Johannesburg, ZAF; 2 Department of Anesthesia, Charlotte Maxeke Johannesburg Academic Hospital, Johannesburg, ZAF

**Keywords:** electromagnetic interference, hemiarthroplasty, deep brain stimulator, general anesthesia, parkinson’s disease

## Abstract

Parkinson’s disease (PD) is a common neurodegenerative disease. The multisystem effects of the disease and its pharmacological treatment have several anesthetic implications. With increasing duration of therapy, pharmacoresistance develops. Deep brain stimulation is a safe and effective treatment for symptom control in advanced PD. Its titratability and reversibility make it an attractive treatment option, and it has replaced surgical ablative procedures for advanced disease management. These devices have several implications in the perioperative period.

A case is presented of a 75-year-old patient for urgent hemiarthroplasty surgery with advanced PD and a deep brain stimulator device in situ.

## Introduction

Parkinson’s disease (PD) is a common neurodegenerative disease affecting 1% of the population older than 65 years of age [1]. The multisystem effects of the disease and its pharmacological treatment have several anesthetic implications [1]. Long-term treatment may lead to the development of pharmacoresistance. Deep brain stimulation (DBS) is a safe and effective treatment for symptom control in advanced PD and has evolved into a clinical standard of care for select patients [2]. Its titratability and reversibility make it an attractive treatment option, and it has essentially replaced surgical ablative procedures for advanced PD management [3].

DBS technology was first approved by the Food and Drug Administration agency in 1996 to treat tremors and essential tremors in PD [4]. Over the last two decades, the application of DBS has expanded to include the management of other movement disorders, certain neuropsychiatric disorders, chronic pain, epilepsy, Alzheimer’s disease, Tourette’s syndrome, and eating disorders [2].

With a greater number of patients being treated with this technology, the anesthesiologist is increasingly likely to encounter patients with a DBS device in theatre or the radiology suite. Unlike pacemakers and implantable cardiac defibrillators where established guidelines exist, there is a paucity of literature pertaining to the perioperative risks and management of DBS devices.

A case is presented of a 75-year-old patient with advanced PD and a DBS device in situ who underwent urgent left hemiarthroplasty surgery. This case was previously presented as an oral presentation at the South African Society of Anesthesiologists Congress meeting in March 2020 in Pretoria, South Africa. 

## Case presentation

A 75-year-old male patient, ASA 2, presented to the emergency department after a fall secondary to loss of balance. There was no history of loss of consciousness. Radiography showed that he had sustained a left femur neck fracture, and urgent hemiarthroplasty surgery was booked.

The patient was known with advanced PD, diabetes, and hypercholesterolemia. He had a history of severe tremors, slow gait, and rigidity that severely affected his activities of daily living. A DBS device was inserted five months prior which improved his functionality. The device had eliminated the tremor, but his gait disturbance and rigidity persisted. His medications included pramipexole (1.5mg twice daily), a combination of carbidopa and levodopa (25mg-100mg thrice daily), metformin (850mg twice daily), aspirin (75mg daily), and rosuvastatin (10mg daily), all of which were not taken on the day of admission. He underwent previous surgeries for insertion of a percutaneous gastrostomy under general anesthesia and DBS device insertion under sedation and general anesthesia, both without any complications.

On examination, the patient looked frail. He had a mask-like face and was mildly dehydrated and slightly pale. His vital signs were as follows: BP 149/80, HR 76, and room air saturation of 93%. The patient's weight was 82kg and his height was 1.8m, with a BMI of 25.3. He had a Mallampati score of II on airway assessment, with a limited cervical range of motion. The DBS internal pulse generator (IPG) was visible under the right clavicle, and a feeding gastrostomy was noted. No abnormalities were found on cardiorespiratory and abdominal examinations. His Glasco Coma Scale scored 15/15, but he had difficulty communicating due to rigidity.

**Figure 1:**
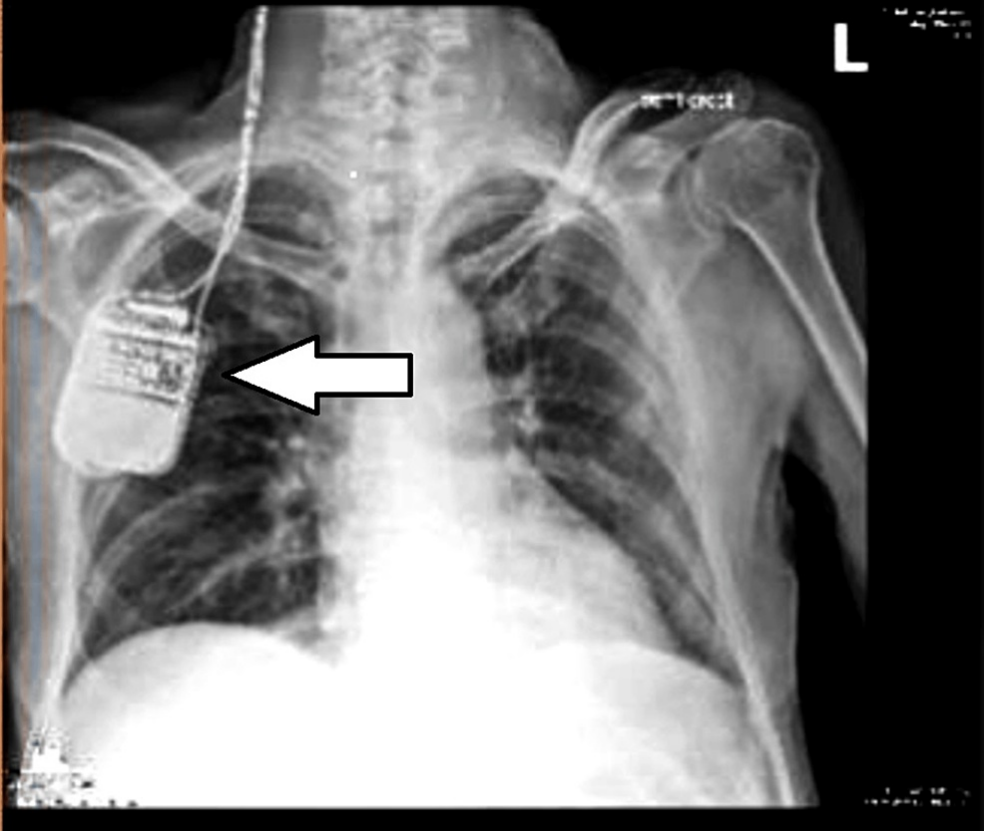
Chest radiography showing the internal pulse generator of the deep brain stimulator device

Blood investigations revealed normocytic normochromic anemia (hemoglobin 10.9 g/dL), normal electrolyte levels, and urea and creatinine of 6.5 mmol/L and 122 μmmol/L, respectively. His chest radiograph (Figure 1) showed the IPG of the DBS device below the right clavicle with extension wires in the neck. His electrocardiograph showed a sinus rhythm with a normal cardiac axis.

The patient was counseled and consented to general anesthesia with a fascia-iliaca compartment block. Prior to surgery, his anti-Parkinson drugs were given via gastrostomy.

In theatre, a fluid bolus of 500ml of warm crystalloid was given prior to induction. General anesthesia was gently induced with fentanyl (150ug), propofol (60mg), and cisatracurium (12mg). An endotracheal tube was placed using videolaryngoscopy, and volume-controlled ventilation was initiated. The DBS device was switched off using the patient control device after induction. A fascia-iliaca compartment block was performed on the left (using 20 ml of 0.375% bupivacaine with 1:200000 adrenalin). A urinary catheter and an arterial line were inserted, both in a sterile fashion. The patient was positioned laterally, and pressure points were protected. A forced air-warming blanket was used, and the temperature was monitored. Cemented bipolar hemiarthroplasty was done using bipolar diathermy, and no adverse events occurred during cement implantation. Arterial blood gases were monitored at induction and before emergence to assess the patient's metabolic profile and to guide fluid resuscitation. The patient experienced labile blood pressure during the surgery (systolic blood pressure range: 81-178 mmHg; mean arterial pressure (MAP) range: 57-124 mmHg). Blood pressure was maintained with intravenous fluid and boluses of phenylephrine initially, and then ephedrine targeting a MAP of >65mmHg. Intravenous paracetamol (1g) and 3mg of morphine were given for analgesia. The blood loss was approximately 400ml.

**Figure 2:**
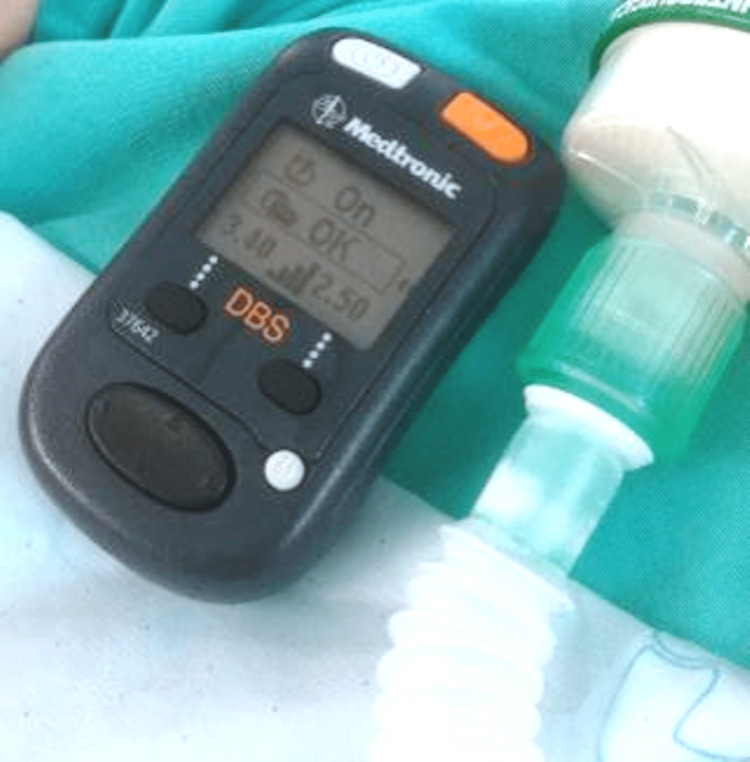
The deep brain stimulator device switched on prior to emergence

Once the procedure was completed, the DBS device was switched on (Figure 2). Neuromuscular blockade was reversed with neostigmine (2.5mg) and glycopyrrolate (0.4mg), and the patient was extubated uneventfully and transferred to the high-care unit. Muscle rigidity persisted after the surgery, but the patient was awake and pain-free. Anti-Parkinson drugs (pramipexole and carbidopa-levodopa) were given timeously via gastrostomy after surgery. On day one postoperatively, the patient had improved muscle tone and was able to communicate without difficulty. He was discharged home two days later.

## Discussion

DBS devices work by suppressing overactivity in the target nuclei in the brain; for most movement disorders, a high suppressive frequency is set (between 130 and 180 Hz) to achieve this [5]. For other conditions, a lower frequency may be set in order to drive activity in the target nucleus. The target nuclei in the brain differ based on the indication [2].

Three components make up the DBS system: the platinum-iridium electrodes covered by a polyurethane sheath, an IPG, and the cable connecting the electrodes to the IPG [2]. The electrode has a quadripolar configuration which allows four stimulating electrodes in a restricted field [2]. Newer IPG devices are smaller and more comfortable [2]. Transcutaneous batteries offer the advantage of longer battery life limiting the need for battery changes and problems related to battery depletion [2]. 

Insertion of a DBS device is a two-stage procedure. During the first stage, intracranial electrodes are inserted into a specific target area in the brain [2]. A stereotactic frame is fixed onto the patient's head and computerized tomography or magnetic resonance imaging (MRI) is performed to precisely localize the target deep brain structures [2,5]. Scalp blocks and conscious sedation are often used to facilitate this stage, but general anesthesia may be required in a patient who cannot tolerate awake surgery [2,5]. The second stage of the procedure involves internalization of the electrodes and insertion of the IPG, typically performed under general anesthesia [2]. The second stage is usually delayed between days to weeks after the first stage but more recently, both stages are performed in the same setting [2].

A few safety considerations must be taken into account in patients with a DBS device in situ presenting for non-DBS-related surgery.

A thorough preoperative assessment should focus on the background medical condition, symptoms, medication, the indication for the DBS device insertion, and symptoms once the device is switched off [6]. Collateral history and records are important if the patient has difficulty with communication. The device must be interrogated preoperatively including the make and model, settings, programmability, battery life, function of the device controller, and previous complications. The neurologist or device technologist may need to be consulted [2,6]. The IPG and pathways of the wires must be assessed on chest radiography so these can be avoided during surgery or nerve stimulation [3]. Rarely, the IPG may be placed in the abdomen [6].

Electromagnetic interference (EMI) can occur perioperatively with the DBS device and ECG waveforms, slow wave diathermy, electrocautery, peripheral nerve stimulators, pacemakers, and defibrillators [2,6]. Diathermy can cause serious injuries and death from heating at the DBS electrode, and thus it is contraindicated [3,7]. Bipolar electrocautery is preferred and should be used in short intermittent bursts. Unipolar cautery and peripheral nerve stimulators can be safely used provided the current path between the cautery electrode and the grounding pad does not traverse the DBS device components [3]. The use of ultrasound, however, obviates the need for nerve stimulation. Puncture sites for regional blocks should not be near the DBS device [3]. In cases where the DBS device is switched off, increased sedation may be needed during regional anesthesia if symptoms are severe [3].

External cardiac defibrillation and cardioversion have been successfully used with a DBS device in situ [3]. It is recommended that the lowest possible output is used, and paddles should be placed far from the device. Implanted cardiac pacemakers and defibrillators can interfere with DBS device and vice versa, by causing inappropriate sensing and responses of the cardiac devices or changes to the DBS device settings [3]. Cardiac devices should be changed to a bipolar sensing mode, and Holter ECG monitoring is needed after any adjustments to the DBS device settings [3]. For patients who have both devices in situ, the IPG of each must be placed away from the other.

To minimize the risk of EMI, the DBS device should be switched off during anesthesia. This can be achieved by holding the patient control device over the IPG unit [5]. Patients with movement disorders or PD should ideally have the device switched off after induction of general anesthesia and switched on before emergence to limit symptoms [2,5,6]. Device deactivation may require changes to the chronic anti-Parkinson medication for symptom control [6]. Gross tremors on emergence or rigidity after extubation has been reported where medication was omitted or delayed [8]. When oral intake is not possible postoperatively, a nasogastric tube may be needed to ensure perioperative continuation of medication. Transdermal rotigotine and subcutaneous apomorphine infusion are alternatives in patients who cannot tolerate enterally [1,8].

Abrupt cessation of anti-Parkinson medication poses the risk of withdrawal syndromes that can manifest in two ways. A rare complication, Parkinson’s hyperpyrexia syndrome, which is caused by withdrawal or withholding of anti-Parkinson medication, has also been described by DBS battery depletion [9] or device malfunction [10]. It mimics neuroleptic malignant syndrome (autonomic instability, pyrexia, and rigidity) and carries a high mortality rate [1]. Neuroleptic agents can similarly cause this syndrome and should be avoided in patients with PD [5]. Dopamine agonist withdrawal syndrome results from stopping of dopamine agonists and has milder symptomatology: anxiety, depression, nausea, orthostatic hypotension, and pain [1].

Drug interactions are important to consider in patients with PD. Propofol can cause dyskinetic movements, but it is nonetheless commonly used and may temporarily eliminate tremors [1,8]. Ketamine and thiopentone have been safely used despite the theoretical risk of PD symptom exacerbation. Volatile agents are safe except for halothane which can exacerbate levodopa-induced arrythmia [1,8]. Induction and volatile agents must be judiciously dosed due to the risk of prolonged hypotension in PD patients with autonomic dysfunction [1]. Neuromuscular blocking drugs are safe but residual blockade can mask PD symptoms [1]. Ideally, reversal of neuromuscular blockade should be assessed using a peripheral nerve stimulator. Opioids have been safely used, but high doses of fentanyl and alfentanil have been associated with rigidity and dystonia, respectively [1,8]. Non-steroidal anti-inflammatory agents can be used in PD to limit opioid use [8]. Anti-emetic agents that cause dopamine antagonism are contraindicated; serotonin antagonists and anti-histaminergic agents are safer alternatives [1,8]. Serotonin syndrome can be precipitated in patients taking monoamine oxidase B inhibitors for PD in conjunction with perioperative administration of other drugs that increase serotonin availability [1,8].

The majority of patients with DBS will need MRI at some point after insertion [7,11]. MRI provides superior images for neurological conditions, and the use of functional MRI technology may have additional advantages. Complications that can arise from MRI include heating at the DBS electrode site, dysfunction of the IPG, induction of current in the DBS hardware, and device movement induced by the magnetic field [7]. Adverse outcomes during MRI, including temporary and permanent neurological injury, have been described in some patients where manufacturer safety recommendations were not strictly followed [3,7,12]. Certain devices are certified MR conditional for full body scans provided that specific recommendations are adhered to, namely: by limiting the coil type, gradient settings, magnetic field strengths, and heating thresholds [7,12,13]. The patient control device, recharger, external neurostimulator, and clinician programmer are not MR-safe [13].

Electroconvulsive therapy (ECT) is safe with a DBS device in situ provided that certain precautions are taken; the ECT electrodes are placed away from the DBS electrodes, the lowest possible threshold for seizures is used and the DBS device is switched off during seizure stimulation [2,3]. Similarly, transcranial motor evoked potential monitoring has been used without complication when the device was switched off [2].

Any potential for EMI requires that the DBS device is switched off during the procedure and interrogated post-procedure [3]. A thorough neurological examination must be documented postoperatively, and the device settings must be adjusted when necessary.

## Conclusions

DBS technology is a standard of care for select patients with advanced PD where pharmacoresistance has developed to conventional anti-Parkinson's drug therapy. The multisystem effects of PD and the effects of the perioperative environment on the DBS device are important considerations in this population group. EMI can occur between the DBS device and monitors or devices in theater. Any potential for EMI warrants that the DBS device be switched off during the procedure, if symptoms permit. Post-procedure, a thorough neurological examination must be documented, and the DBS device should be interrogated. 
